# On the Climate of California in Its Relation to the Treatment of Pulmonary Consumption

**Published:** 1861-02

**Authors:** James Blake


### ON THE CLIMATE OF CALIFORNIA

### IN ITS RELATION TO THE TREATMENT OF PULMONARY CONSUMPTION.

BY JAMES BLAKE, M. D., F. R. C. S.

In my previous article on the subject of phthisis, I have shown that both an improved pathology and the result of experience derived from a vast mass of facts, point to the open air plan of treating the disease as that most likely to cure our patients. So strong is my faith derived from my own experience, in its efficacy for curing the disease, that I believe fully seventy-five per cent. of cases taken in hand in the second stage of the disease, can not only be temporarily relieved, but permanently cured, by a rationally conducted open air treatment, and that at least fifty per cent. of the cases that have advanced to the third stage of the disease, or in which cavities have already begun to form, can be cured, and in nearly all of them can life be prolonged by the same treatment.* The open air treatment, however, like every other plan of treatment for the cure of disease, has, in order to be successful, to be carried out in a rational manner. There are many obstacles to be overcome, many modifications to be made in it, in order to render it available for the different classes of our consumptive patients. As civilization has advanced, we have departed more and more from the nomadic life of our forefathers, and

*This estimate, as to the curability of the disease, is far below that which my own limited experience has furnished, but the cases in which the treatment has been carried out, have been too few to justify me in drawing any definite conclusions as to the proportion of cases that can be cured. I would merely state that, in three cases with cavities in the lungs, (in one case on each side,) in which the treatment has been pretty fairly carried out, one case is cured and has remained free from cough during the last two years; another case, in which there were cavities in each lung, now reports himself well, has hardly any cough for the last twelvemonth, and has regained his usual weight; and a third case, a most unpromising one, is much improved, having gained 10 pounds in weight during the last summer, and the cavity having ceased to suppurate.

our social organization as at present constituted, may seem to many to present insurmountable difficulties to carrying out the treatment, particularly when it has to be applied to our female patients. I trust, however, to show that these obstacles can be overcome with much less difficulty than would at first sight be supposed by those accustomed to live only amidst the artificial luxuries and comforts of our in-door glass-window hot-stove civilization, particularly when it can be demonstrated to our consumptive patients that these very luxuries and comforts are dragging them to an early grave, to which they can but serve to smooth the path. Could we have a model climate, that is one in which the temperature should never fall below 45°, and in which it never rained, the best advice we could give our patients would be to live entirely in the open air, where every breath they would inspire would be taken in, fresh and uncontaminated, from the aereal ocean. Under these conditions, and under these conditions only, can the maximum of that health-restoring influence which is desirable fron fresh air, be obtained. But as climates were not specially made for the treatment of consumptive patients, we must do the best we can with them as they are. Even in California, although our climate affords as many facilities for carrying out the open air plan of treatment as any in the world, yet it necessitates us to place our patients occasionally in positions where they are obliged to breathe portions of the same air a second time. Again, a large proportion of our patients are so situated as regards their business, or their means of obtaining a livelihood, that they are unable to completely separate themselves from their affairs, to lead a nomadic life, even during the time that the season would permit of their so doing. Supposing, however, that our patient is in a position to follow our advice fully, and presents himself to us in the second or third stage of the disease, before too large a portion of the lung is completely disorganized, or the strength too far exhausted. My advice to him is, between the months of May and October, immediately to assume the nomadic life; to get on horseback with a pack mule and with a companion that can hunt, break away for some part of the coast range, where he can command an elevation of two or three thousand feet, and where game is plenty. It signifies little to what part of the range he directs his steps, or he may choose the Sierra, if more convenient. I consider, however, the coast range preferable, for many reasons. The climate there is more agreeable; the heat of the day is not so great, nor are the nights so cold early and late in the summer as on the higher hills of the Sierra. Game is much more abundant on the coast range than on the Sierra, and a good and wholesome nourishment can thus be more readily obtained. Another advantage that the coast range has over the Sierra is, that our patients, when once they are well up in the coast range, have not such facilities for rushing back to the "*flesh-pots of Egypt*" as they have in the Sierra, with most parts of which there is daily stage communications with the valleys. It is a great advantage for them to be obliged to stay out, but it requires much strength of mind, or some pretty steep barriers, to prevent a man who has a family from seizing the first opportunity afforded by a partial return of health, for revisiting his home, or for a man who is in business, form satisfying himself as to the way in which his affairs are being carried on. I have seen much mischief done by these too frequent visits at home, particularly in the case of married men, who the more they are away from their wives the better. I consider, that, during the summer months, our patients in this latitude will find the best climate at an elevation of from two to four thousand feet above the level of the sea. The lower elevation is the best in early summer and autumn, and can be borne by patients whose lungs are considerably diseased, whilst the higher elevation will suit those in whom the third stage has not made much progress. There is an opinion generally prevalent amongst the members of the profession, that phthisical patients do better at lower levels, on account of the air not being so much rarified, and therefore requiring a less respiratory effort to obtain the same amount of oxygen. My experience is entirely opposed to this idea of high elevations not suiting phthisical patients, as is also that of many other physicians who have practiced in mountainous regions. Except in cases in which a considerable portion of both lungs is diseased, I believe that the additional expansion required to obtain the necessary supply of oxygen, may even be beneficial. But we have a far more important reason for sending our patients to the mountains than that afforded by any problematical advantage to be gained by this additional expansion of the lung, and this is, in the decided superiority of the mountain air over that of the valleys, for invigorating the digestive organs. There can be no comparison between the two climates in this respect, and as phthisis is to be attacked and cured through these organs, a mountain climate is always to be preferred. There cannot be a more striking instance of the impunity, as regards the respiratory organs, with which great elevations can be borne by phthisical patients, than is furnished by Dr. Smith, of Lima, in an article "On the Climate of Peru," in the 18th vol. of the *British and Foreign Medico-Chirurgical Review.* After stating the symptoms of the second stage of phthisis, as generally seen in that country, Dr. Smith observes: "No Lima junta of experienced native, or well acclimated European physicians, would for a moment hesitate to order to the Sierra a patient in the condition I have just described. They would deem this transfer of climate, as the only security for the patient. Under such conditions I have witnessed the application of all approved European remedies of every school fully tried, where the phthisical patient was, for one reason or another, destined to run his course on the coast and in the capital, under the eye of able assistants, but always with the same fatal termination."

In answer to the query as to the best locality for the treatment of phthisis, he observes: "On the Pacific slope of the Cordillera, and by the Pasco road from Lima, Haraway and Canta are considered the best localities, but Canta, above all, on this route, is allowed to be the most desirable, being about twenty-five leagues from Lima, and at an elevation of 10,000 feet. Again by the Zarma road from Lima, Matucana and San Mateo are favorable climates—the former, according to McLean, is 8,026, and the latter 10,984 feet high." After this, it is quite useless for us to prevent our patients seeking a mountain climate, for fear of rarified air. In our latitude a much less elevation will suffice to ensure a good digestive climate than in a country so near the Equator as is Peru. The great desideratum is, to find a climate where the digestive organs can recuperate, and the lungs, if not very far gone, can afford to go and seek it at elevations of 10,000 feet, if necessary. When phthisis was looked upon simply as a disease of the lungs, and its pathology was sought solely in the changes taking place in these organs, it is not surprising that physicians should hesitate to send their patients to elevations, where the diseased organ should have more work to perform. But now that more correct views prevail as to the cause of the disease, there can be no longer any excuse for us preventing our patients from seeking the best localities for their recovery, by migrating to the mountains.

There is another advantage possessed by our mountain climates, as compared with that of the valleys, which is, that during the warmer summer months the diurnal variations are less than in the valleys. When residing at Iowa Hill, at an elevation of about 4,000 feet, I found, on comparing my meteorological register with that kept by Dr. Hatch, in Sacramento, that when the thermometer in the valley would fall 16° to 20° between sunset and sunrise, it would only fall 10° to 14° at Iowa Hill; nor would the temperature reach so high a point during the day—the daily range of temperature being 10° to 12° less at the higher station than at the lower. Another great advantage that our patients have by residing in the mountains during the summer months is, that they avoid malaria. The extraordinary opinion that once prevailed, as to the antagonism between malarious disease and phthisis, is now fortunately exploded. Amongst the vast mass of medical fallacies that have been at different times advanced, it would be difficult to find one with less foundation. According to my experience, malaria is extremely prejudicial to phthisical patients. They are very liable to be affected by it, and its effects are amongst the most unfortunate complications that can interfere with the cure. The effects are seldom seen in the form of an intermittent. They generally assume some irregular type, so that it is frequently difficult to refer them to their true cause.

I trust these facts and considerations will suffice to convince the profession that a mountain climate, at least such as we possess in the coast range and the Sierra, affords the most favorable localities in which a phthisical patient can pass the summer and autumn months. Cases sometimes present themselves in which elevations above one or two thousand feet cannot be borne without oppression; they are, however, rare, and are, I think, depending on some asthmatic affection. During the winter our patients, where practicable, may derive benefit from a trip to the Sandwich Islands, provided they do not suffer too much from sea-sickness.

The months of December and January can be thus well spent, and these months are undoubtedly the worst months in the year for consumptive patients in California. If they make the voyage, let them not remain on the Islands; the benefit is to be derived from the voyage, not from a residence on the Islands, where the moist, warm atmosphere is not calculated to improve the digestive organs. It is by a dry, bracing atmosphere that these are usually invigorated, although a sea-voyage, in an atmosphere almost saturated with humidity, often exerts a favorable influence in restoring the digestive functions. It is only on the sea, however, or on the coast, that such an atmosphere can be borne with impunity; as soon as we get inland, humidity is undoubtedly prejudicial, so that the benefit to be derived from a sea-voyage must be sought for in some other element of the sea-air, besides its moisture. Should it not be desirable, however, for our patients to leave the State, Los Angelos, Santa Cruz, or San Diego, are good localities for passing the winter, or even the lower hills of the Sierra or coast range. At an elevation of a few hundred feet, the sea of fog that frequently fills the valleys for days together during the months of December and January, seldom rises, and our patients can thus secure many days of bright sunshine, which in the valleys would have to be passed in a cold, bone-searching fog. During the months of February, March and April, San Francisco, or the inland valleys, afford about as good localities for our patients as any in the State; there is no fog, no dust, no malaria, and the climate is more equable than that of the mountains at the same season.—*Pacific Med. and Sur. Journal.*

